# A Review of the Relationship between Lentil Serving and Acute Postprandial Blood Glucose Response: Effects of Dietary Fibre, Protein and Carbohydrates

**DOI:** 10.3390/nu14040849

**Published:** 2022-02-18

**Authors:** Sandra T. Clarke, Sidra Sarfaraz, Xinye Qi, Davin G. Ramdath, Gregory C. Fougere, D. Dan Ramdath

**Affiliations:** Guelph Research and Development Centre, Agriculture and Agri-Food Canada, Guelph, ON N1G 5C9, Canada; sandra.clarke2@agr.gc.ca (S.T.C.); sidra9518@gmail.com (S.S.); katy.qi@agr.gc.ca (X.Q.); davinramdath@gmail.com (D.G.R.); gcfougere@uwaterloo.ca (G.C.F.)

**Keywords:** lentil, glycemic response, insulin, protein, dietary fibre, carbohydrates, human trials

## Abstract

Pulse consumption has been shown to confer beneficial effects on blood glucose and insulin levels. Lentil consumption, in particular, consistently lowers acute blood glucose and insulin response when compared to starchy control foods. The mechanism by which lentils lower postprandial blood glucose response (PBGR) and insulin levels is unclear; however, evidence suggests that this effect may be linked to macronutrients and/or the amount of lentils consumed. This review attempts to consolidate existing studies that examined lentil consumption and glycemic and/or insulinemic responses and declared information on macronutrient composition and dietary fibre content of the foods tested. Collectively, these studies suggest that consumption of lentils reduces PBGR, with the minimum effective serving being ~110g cooked to reduce PBGR by 20%. Reductions in PBGR show modest-to-strong correlations with protein (45–57 g) and dietary fibre (22–30 g) content, but has weaker correlations with available carbohydrates. Increased lentil serving sizes were found to moderately influence relative reductions in peak blood glucose concentrations and lower the area under the blood glucose curve (BG AUC). However, no clear relationship was identified between serving and relative reductions in the BG AUC, making it challenging to characterize consistent serving–response effects.

## 1. Introduction

Type 2 diabetes (T2D) is characterised by impaired fasting blood glucose (BG) levels and is associated with increased risk for coronary heart disease, stroke, and a two-fold increase in overall mortality [1]. Chronic elevation of BG was also linked to obesity, hypertension, and coronary heart disease, even in persons without a history of diabetes [1]. As such, the Canadian clinical practice guidelines for diabetes stresses the importance of dietary approaches, including the increased use of pulses, for achieving optimal glycemic control in persons with T2D [2,3]. Lentils are dry harvested grains containing significant amounts of resistant, slow and rapidly digestible starches, with a range of protein depending on the variety [4]. These pulses also contain many polyphenolic compounds, with a majority being flavonoids, that contribute to health-promoting properties such as antioxidant activity and delayed glucose and lipid digestion, which could contribute to improved glycemic control and reduced risk for obesity [5]. Incorporation of pulses into daily meal consumption was shown to improve glycemic control partly due to their low glycemic index (GI), and this effect may vary between lentil varieties [6,7,8,9]. For example, a meta-analysis of non-oilseed pulse intervention studies showed that regular consumption of chickpeas, beans, peas and lentils improved glycemic control in both healthy and diabetic participants [9]. However, lentil dosage varied significantly amongst the various studies, with a major problem being that dry and wet weights are not regularly reported in published articles. Varying reductions in area under the blood glucose response curve (BG AUC) were observed in study participants who consumed lentils ranging from as much as 715 g per serving [10] to as little as 50 g [11]. As such, the minimum effective serving of lentil required to significantly and consistently lower BG has not yet been determined. An important feature of the latter effect is that this amount of lentils should be easily consumed by the average adult. In addition, there is a need to ascertain whether a serving–response relationship exists between lentil consumption and BG lowering. At present, it is unclear whether consuming larger quantities of lentils results in a larger attenuation effect on glycemic response or improved glycemic control.

The BG-lowering effects of lentil consumption have been independently attributed to carbohydrate composition and protein content [9]. In a regression analysis of pulse consumption in low-GI or high fibre diets, dietary carbohydrates accounted for 22% of the variation in fasting blood glucose (FBG) while proteins accounted for 14% [9]. However, this analysis also found that the quantity of either protein or carbohydrate did not differentially alter the effect of non-oil-seed pulses on glycosylated blood proteins or FBG [9]. It was suggested that the source of carbohydrates used in control foods (starch, glucose or sucrose) is the most important factor in determining whether a protein-containing treatment is capable of impacting BG and insulin responses in nondiabetic participants [12]. Furthermore, in that analysis, up to 40% of the variation in fasting blood insulin concentrations could be explained by fiber content of pulses [9]. In contrast, results of meta-analyses suggest that the BG-lowering effects of pulses were not attributed to fibre content; rather, pulses independently reduced FBG and glycosylated proteins in T2D patients [9,13]. More recently, ingestion of β-glucan, a soluble fibre, was reported to reduce postprandial BG AUC and insulin levels in healthy and diabetic individuals [6]. Clearly, the contribution of dietary fibre, protein and carbohydrates towards the attenuation of postprandial BG AUC is not fully understood. It is important that this relationship be delineated for regulatory purposes, and to guide the manufacture of pulse and lentil-based functional foods.

The objectives of this review are: (i) to attempt to establish a minimum effective serving for lentils to lower BG concentrations; (ii) to determine whether there is a serving–response relationship between the amount of lentils consumed and PBGR, and (iii) to attempt to identify the dietary component responsible for the glucose-lowering effect of lentils. 

## 2. Materials and Methods

This review examined data from published research on effects of lentil consumption on both diabetic and non-diabetic participants. A systematic search and reporting of data was carried out in accordance with the Preferred Reporting Items for Systematic Reviews and Meta-Analyses (PRISMA) guidelines [14]. 

### 2.1. Study Selection

Literature searches were conducted using PubMed, Scopus, CABI Global Health, Google Scholar, Canadian Agricultural Library, and the University of Guelph Library databases for relevant studies published from 1980 until 4 March 2021. The following search terms and Boolean operators were used: “lentil AND blood glucose AND diabetes AND postprandial AND acute”. In order to retrieve published studies involving healthy participants, the term “AND diabetes” was removed. The search was restricted to acute human studies; those that measured only chronic responses to lentil consumption or concurrent interventions with other pulses were excluded. All studies included in this analysis needed to have had BG and insulin concentrations measured within the first two hours following lentil consumption, and these measures were defined as an “acute” or “postprandial” response. 

### 2.2. Data Extraction

The relevant data, study characteristics and outcomes were independently extracted by three investigators (S.S., D.G.R., G.C.F.). The total amount of lentils consumed by participants in each study was recorded as the cooked weight. Raw weights provided by these studies were converted to cooked weights using the average moisture content of green lentils (68.4%), as gathered from our previous research (unpublished data). The cooked weight was calculated as follows: (dry weight/% solids) × 100, where % solid = 100–68.4. Macronutrient profiles including protein, available carbohydrates (CHOav) and total carbohydrate, as well as dietary fibre content of the lentil treatments were also recorded, if included in the publication. If total carbohydrate data were not provided, it was calculated by adding CHOav and dietary fibre values. Time and value of the maximum BG concentration (BG Cmax) were recorded for lentil and control treatment groups. Glycemic response or AUC, was also extracted, if available; if not, absolute differences and percent reductions in AUC between lentil and control treatments were calculated. Insulin data, maximum insulin concentration (insulin Cmax), area under the blood insulin response curve (iAUC), and the absolute difference between peaks were also recorded.

### 2.3. Data Analysis

Relationships between glycemic response and dependent variables of lentil serving size, protein, dietary fibre, and total carbohydrates were explored using linear regression and multiple regression analyses. Linear, exponential, logarithmic, and quadratic correlations were assessed. An appropriate line of best fit for each specific outcome was chosen by comparing the correlation coefficient and standard error of the mean, as well as a visual inspection of each plot. From these assessments, AUC was noted as either an absolute value or relative reduction. If not provided in the study, relative reductions in AUC were calculated manually by using [(AUC_CONTROL_ − AUC_LENTIL_)/AUC_CONTROL_] × 100%. For studies that provided AUC in different units (e.g., mg × h/dL), measurements were converted to mmol × min/L by standard conversion factors (e.g., 1 mg/dL glucose = 0.05551 mmol/L glucose). For studies that represented data only in graphical formats, Plot Digitizer (Ver. 2.6.8; Source Forge; accessed on 7 January 2022; http://plotdigitizer.sourceforge.net) was used to digitise functional data from plots, and the average of three estimates for each data point was used to obtain the reported value. Graphing and data analysis was carried out using GraphPad Prism (Ver. 9.2; GraphPad Software, San Diego, CA, USA). The Pearson coefficient (*r*) was calculated for linear relationships, while Spearman’s rank correlation coefficient (*r_s_*) was used for describing non-linear relationships. A value of *p* < 0.05 was considered to be indicative of a significant relationship.

## 3. Results

A total of twelve studies that measured BG and/or insulin responses to various lentil treatments in healthy participants were identified (Table 1), and an additional six studies involved diabetic participants (Table 2). However, several studies did not provide all the variables relevant to this review, such as the lentil serving size and the amount of CHOav. Some studies only provided BG concentrations, and not the AUC. In addition, six studies included data on insulin; four with healthy participants and two with diabetic participants. While there were insufficient number of studies to clearly define correlations, some trends between lentil serving and the corresponding beneficial impacts on AUC and insulin responses were observed in healthy participants.

### 3.1. Lentil Serving Size

**Figure 1:**
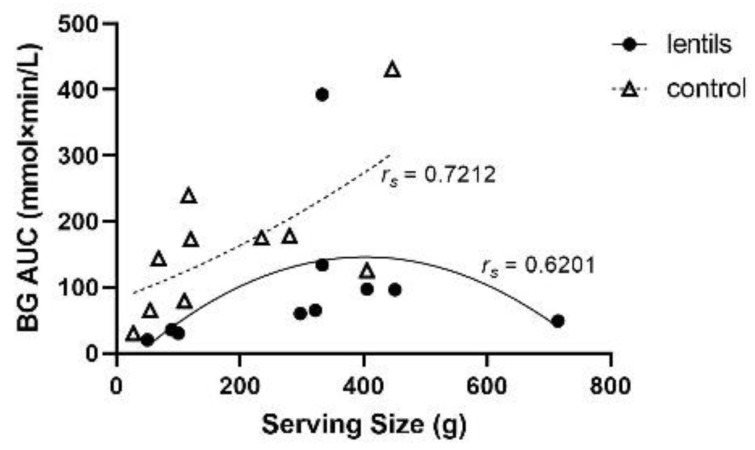
Area under the blood glucose response curve (BG AUC) for various lentil and control serving sizes (g). A quadratic model best fit the lentil (*r_s_* = 0.6201; *n* = 10; *ns*) and control data (*r_s_* = 0.7212; *n* = 10; *p* < 0.05).

**Figure 2:**
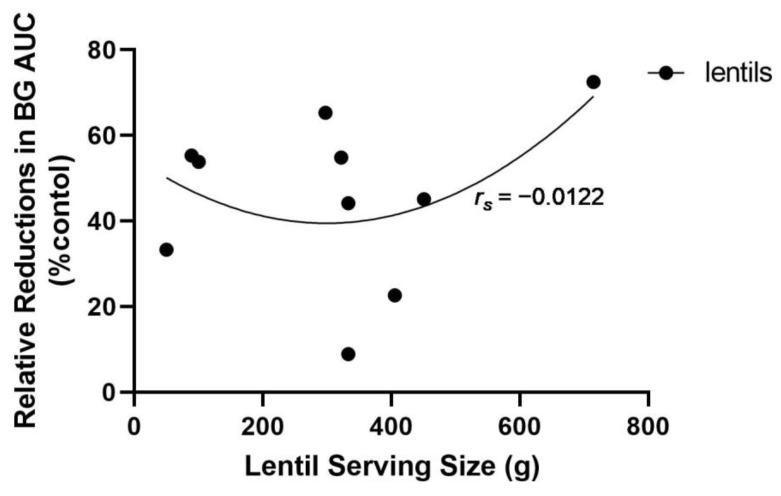
Relative reductions (%) in area under the blood glucose response curve (BG AUC) according to lentil serving sizes (g) with reference to controls. Relative reduction was calculated using the formula: ((Lentil BG AUC–Control BG AUC)/Control BG AUC) × 100. A quadratic curve best fit this data (*r_s_* = −0.0122; *n* = 10; *ns*).

Among healthy participants, BG AUC displayed a quadratic relationship with increasing lentil serving size (Figure 1). As control treatment servings increased, BG AUC tended to increase at a nearly exponential rate; this was expected given that there would be a concomitant increase in CHOav. At nearly equivalent servings, BG AUC tended to be lower with lentil treatments than controls. For lentil treatments, AUC increased at a slower rate compared to controls, reached a lower peak and began to trend towards a decrease in AUC at higher servings. A quadratic model best fitted the lentil (*r_s_* = 0.6201; *n* = 10; *ns*) and control data (*r_s_* = 0.7212; *n* = 10; *p* < 0.05) for BG AUC according to serving size. As such, the minimum effective serving of cooked lentil for reducing blood glucose AUC appears to be between 100 and 120 g. There did not appear to be an association between lentil serving size and relative reduction in AUC compared to control foods (Figure 2); however, servings between 100 and 120 g lentils resulted in relative reductions of BG AUC that were similar to servings of between ~350 and 450 g. The large variation in the relative reduction in AUC by lentil at servings between 300 and 500 g is suggestive of the need for more carefully controlled acute feeding trials involving human participants. 

### 3.2. Protein

**Figure 3:**
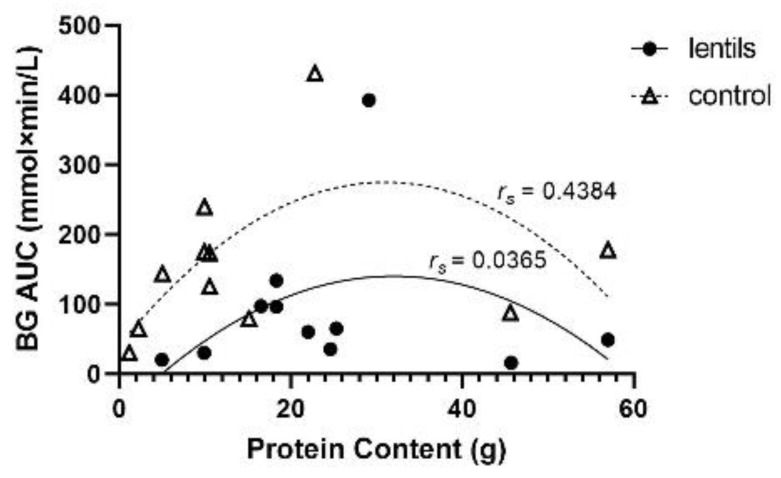
Relationship between area under the blood glucose response curve (BG AUC) and protein content (g) in lentil treatments and controls. Quadratic models for both lentil (*r_s_* = 0.0365; *n* = 11; *ns*) and control treatments (*r_s_* = 0.4384; *n* = 11; *ns*) best fit the data.

Analysis of the available data suggest that there may be a quadratic relationship between protein content of test meals and BG AUC, with both low and high protein intakes being associated with lower BG AUC (lentil *r_s_* = 0.0365; *n* = 11; *ns*; control *r_s_* = 0.4384; *n* = 11, *ns*; Figure 3). At higher protein content values, BG AUC was lower for both lentil treatments and controls, although lentil treatments resulted in lower BG AUC at similar protein content of control foods. The relationship between increased protein content and lower BG AUC within lentil treatments followed a similar quadratic trend to that of the BG AUC lowering effect noted with increased lentil serving size. However, it is unlikely that protein content is the sole determinant of BG AUC reduction associated with lentil consumption; it appears that portion size may contribute to this effect. As an example, a study that examined a lentil-based breakfast with 57 g of protein in a 387 g total meal serving size resulted in a mean (±SEM) BG AUC of 49 ± 13 mmol × min/L, whereas the control meal with the same protein content and 643 g meal serving size produced a BG AUC of 178 ± 34 mmol × min/L [10]. A similar finding was observed when two studies with similar protein content were compared. The first study investigated a 50 g serving size of green lentils containing 4.94 g of protein, and this led to a BG AUC of 20 ± 5 mmol × min/L [11]. The second study used a control diet of instant potato flakes containing 5.0 g of protein with 50 g of equivalent CHOav, and this led to a BG AUC of 268 ± 40 mmol × min/L [15]. Despite treatments being nearly equivalent in protein content, the BG AUC of participants consuming lentil treatments were notably lower than that of the control treatments. The amount of total protein and serving size both appear to have notable impacts and may each have important roles in the BG AUC lowering effect of lentil treatments. 

### 3.3. Dietary Fibre

**Figure 4:**
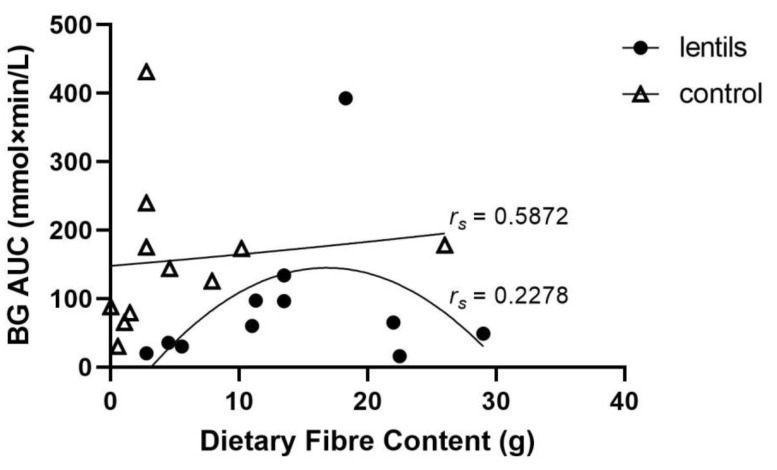
Area under the blood glucose response curve (BG AUC) according to dietary fibre content (g) in lentil treatments and controls. Lentil data best fit a quadratic model (*r_s_* = 0.2278; *n* = 11; *ns*), and control data followed an exponential trend (*r_s_* = 0.5872; *n* = 11; *ns*).

Lentil treatments generally had more dietary fibre content compared to corresponding controls, and this was associated with lower BG AUC values (Figure 4). Lower BG AUC values (<100 mmol × min/L) were reported at both low (<10 g) [16] and high levels (>20 g) [10,15,17] of fibre, following a trend similar to that of protein content. While BG AUC initially increased with increasing fibre content, it quickly reached a peak and started to decrease as the fibre content of the treatments increased. The BG AUC followed a quadratic model for protein content of lentil treatments (*r_s_* = 0.2278; *n* = 11, *ns*), while controls followed an exponential trend (*r_s_* = 0.5872, *n* = 11, *ns*).

### 3.4. Available Carbohydrates (CHOav)

**Figure 5:**
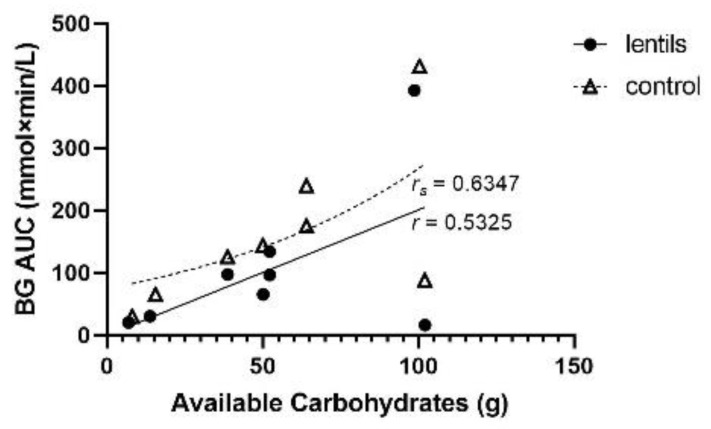
Area under the blood glucose response curve (BG AUC) response at various amounts of available carbohydrates (CHOav) in the lentil treatment or control. A linear model best fit the lentil data (*r* = 0.5325; *n* = 8; *ns*), and the control data followed an exponential trend (*r_s_* = 0.6347; *n* = 8; *ns*).

The relationship between BG AUC and CHOav appeared to be linear for lentil treatments (*r* = 0.5325, *n* = 8; *ns*; Figure 5), while controls followed an exponential trend (*r_s_* = 0.6347; *n* = 8; *ns*). For both lentil treatment and control groups, an increase in CHOav provoked a progressive increase in AUC. In general, participants who consumed lentil treatments had lower BG AUC values compared to controls prepared from starchy foods (potatoes, bread, or pasta), and this trend was apparent at all levels of CHOav. Even at low levels of carbohydrate intake such as that observed in a study with green lentils (6.91 g CHOav), the BG AUC was significantly lower (20 ± 5 mmol × min/L) than a white potato control with 6.14 g of CHOav (BG AUC 42 ± 5 mmol/L × min, *p* < 0.001) [11]. 

**Figure 6:**
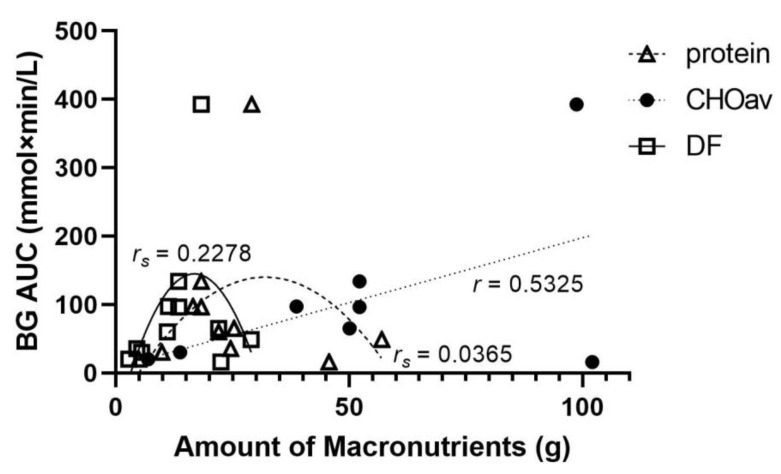
Area under the blood glucose response curve (BG AUC) according the amount of macronutrients or dietary fibre (g) per lentil treatment serving. This plot combines the lentil data of Figure 3, Figure 4 and Figure 5. Quadratic modeling best fit protein (*r_s_* = 0.0365; *n* = 11; *ns*) and dietary fibre (DF) (*r_s_* = 0.2278; *n* = 11; *ns*), and a linear model best fit available carbohydrates (CHOav; *r* = 0.5325; *n* = 8; *ns*).

**Figure 7:**
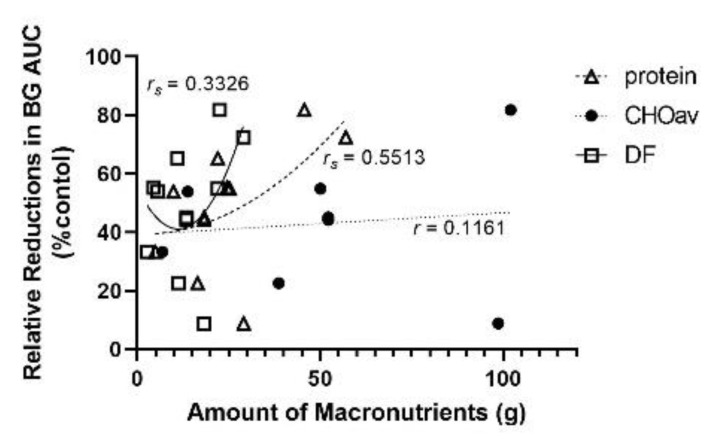
Relative reductions (%) of area under the blood glucose response curve (BG AUC) according to the amount of macronutrients or dietary fibre (g) in the lentil treatment group with respect to the control group. Quadratic modeling best fit protein (*r_s_* = 0.5513; *n* = 11; *ns*) and dietary fibre (DF; *r_s_* = 0.3326; *n* = 11; *ns*), and a linear model best fit available carbohydrates (CHOav; *r* = 0.1161; *n* = 8; *ns*).

When macronutrients (protein, CHOav) and dietary fibre in the lentil treatments were compared separately, all showed positive correlations with BG AUC, although the correlation coefficients were relatively small (Figure 6): CHOav had a moderate linear relationship (*r* = 0.5325; *n* = 8; *ns*), while a quadratic model best fit protein (*r_s_* = 0.0365; *n* = 11; *ns*) and dietary fibre (*r_s_* = 0.2278; *n* = 11; *ns*). The magnitude of relative reduction of BG AUC decreased with increasing levels of CHOav and dietary fibre (Figure 7). The BG AUC reduction with lentil treatment relative to control was greatest with increasing protein content in a quadratic model (*r_s_* = 0.5513; *n* = 11, *ns*). Although moderate, this correlation was greater than that between BG AUC relative reduction and CHOav (*r* = 0.1161; *n* = 8; *ns*; linear model) or dietary fibre (*r_s_* = 0.3326; *n* = 11; *ns*; quadratic model). 

### 3.5. Maximum Blood Glucose Concentrations

**Figure 8:**
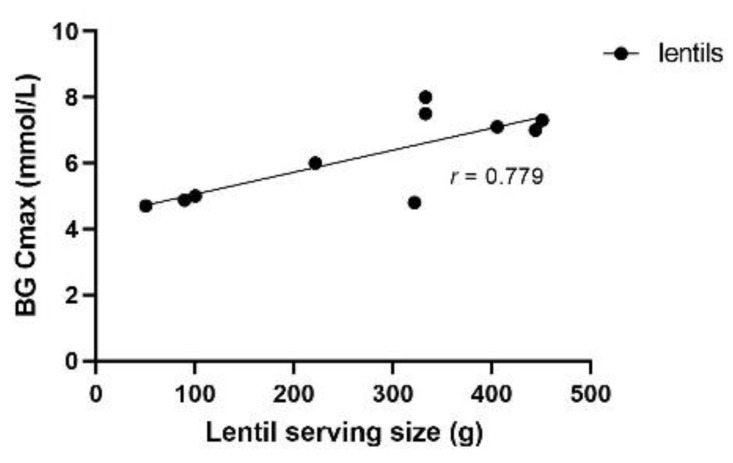
Maximum BG concentration (Cmax, mmol/L) according to lentil serving size (g). The linear trend best represented data (*r* = 0.779; *n* = 10; *p* < 0.01). The largest serving size investigated (715 g) was removed from this plot.

**Figure 9:**
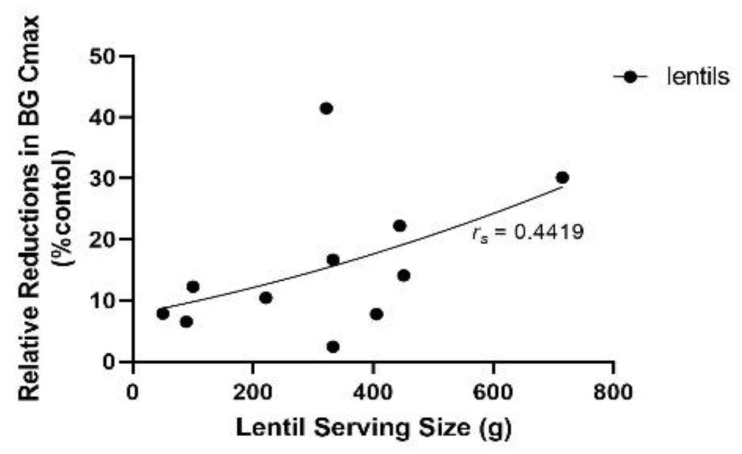
Relative reductions (%) of blood glucose maximum concentration (BG Cmax) according to the lentil serving size (g) in the lentil treatment group with respect to the control group. A quadratic model best fit this data (*r_s_* = 0.4419; *n* = 11; *ns*).

BG Cmax also followed a quadratic trend with lentil serving size (*r_s_* = 0.5968, *n* = 11, *ns*). BG Cmax was not remarkably different when high and low lentil serving sizes were compared, as values of 4.7–5 mmol/L were obtained with sservings as low as 50 g and as high as 715 g of cooked lentils (Table 1; Figure 8). The latter serving size appeared to skew this relationship; when removed there was a significant linear relationship between BG Cmax and lentil serving (*r* = 0.779; *n* = 10; *p* < 0.01). The relative reduction in BG Cmax also had a quadratic correlation with lentil serving size (*r_s_* = 0.4419; *n* = 11; *ns*; Figure 9); as the serving size increased, so did the relative reduction in BG AUC compared to controls. 

### 3.6. Macronutrients, Dietary Fibre and BG Cmax

**Figure 10:**
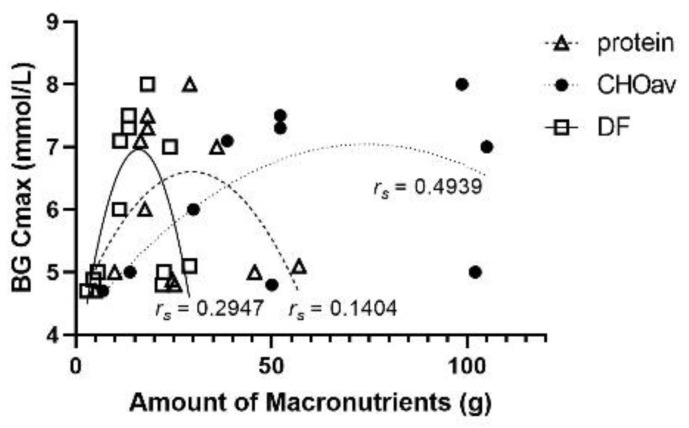
Maximum BG concentration (Cmax, mmol/L) according to the amount of macronutrients or dietary fibre (g) in the lentil treatments. Quadratic modeling best fit each component; protein (*r_s_* = 0.1404; *n* = 12; *ns*), dietary fibre (DF; *r_s_* = 0.2947; *n* = 12; *ns*) and available carbohydrates (CHOav; *r_s_* = 0.4939; *n* = 10; *ns*).

**Figure 11:**
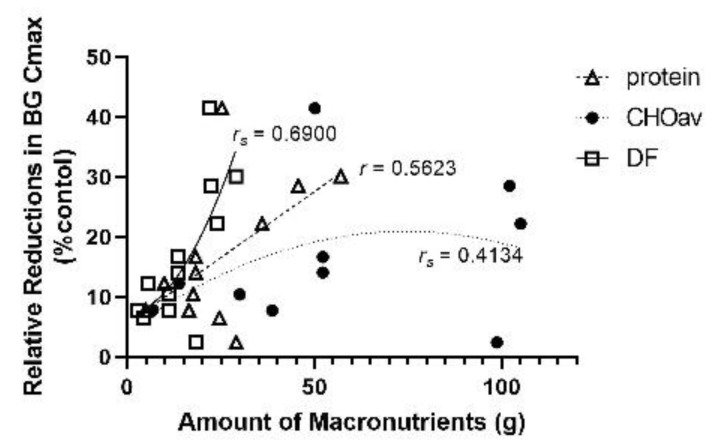
Relative reductions (%) of maximum BG concentration (Cmax, mmol/L) according to the amount of macronutrients or dietary fibre (g) in the lentil treatment group with respect to the control group. Available carbohydrates (CHOav; *r_s_* = 0.4134; *n* = 10; *ns*) and dietary fibre (DF; *r_s_* = 0.6900, *n* = 12; *p* < 0.05) were best described with quadratic modeling, and protein (*r* = 0.5623; *n* = 12; *ns*) was best fit with a linear model.

Protein (*r_s_* = 0.1404; *n* = 12; *ns*), CHOav (*r_s_* = 0.4939; *n* = 10; *ns*) and dietary fibre (*r_s_* = 0.2947; *n* = 12; *ns*) all displayed quadratic relationships with BG Cmax (Figure 10); however, their relative abundance in test meals varied, with dietary fibre having the lowest and CHOav having the highest range of values. Dietary fibre had a weak correlation with BG Cmax, and increased dietary fibre content in the test meals was associated with reduced BG Cmax, even at low levels of intake. With respect to relative reduction of BG Cmax (% control), CHOav (*r_s_* = 0.4134; *n* = 10; *ns*) and dietary fibre (*r_s_* = 0.6900; *n* = 12; *p* < 0.02) both displayed quadratic relationships, while protein demonstrated a linear relationship (*r* = 0.5623; *n* = 12; *ns*; Figure 11). The relationship between BG Cmax reduction and dietary fibre was significant, and both dietary fibre and protein content had relative reductions in BG Cmax with increased content. On the other hand, the impact of CHOav content on relative reductions in BG Cmax was inconsistent across studies. A maximum relative reduction in BG Cmax (41.5%) was achieved at 50 g CHOav, and relative reductions in BG Cmax of 22.2% and 28.5% were recorded from studies containing 105 g and 102 g of CHOav respectively. In contrast, there was only a minimal relative reduction in BG CMax (>3%) in a study that contained 98.7 g of CHOav in the lentil meal. 

### 3.7. Studies Involving Diabetic Participants

Trends were more difficult to elucidate with diabetic participants as most studies included in this review did not provide BG AUC. Although it is usually expected that persons with diabetes have an exaggerated glycemic response, the relative difference in BG AUC and BG Cmax should give some indication of the efficacy of lentils. Upon assessment of the studies listed in Table 2, the available data show that relative reduction in BG AUC, following lentil treatments, ranged from approximately 24% to 68%, which is a clinically significant effect. Further, it is clear that lentil treatments also resulted in reduction of BG Cmax of between 20% and 76%, with the mean (SD) relative reduction being 51.7 ± 25.4%.

### 3.8. Insulin

In general, most studies did not report insulin data following lentil consumption; however, we identified six studies with insulin data: four studies included healthy participants and two with diabetic participants (Table 3). In healthy participants, the maximum concentration of insulin (Cmax) was 50 pmol/L after the consumption of a 100 g cooked green lentil treatment containing 9.9 g protein, 5.6 g fibre, and 26.3 g total carbohydrates. This represented a relative reduction in insulin Cmax of 37.5% at 15 min following consumption compared to control [11]. In another study with healthy participants, a considerably larger serving of cooked green lentils (715 g) led to a 56.5% relative reduction in insulin Cmax at 60 min compared to the control [10]. In diabetic participants, 225 g of cooked green lentils containing 20.5 g of protein was associated with an insulin Cmax of 216 ± 66 pmol/L at 100 min [18], and similarly a 297 g serving of cooked lentils with 22.4 g of protein led to an insulin Cmax of 174 ± 6 pmol/L at 120 min [19]. These two studies required T2D participants to consume 50 g of carbohydrates in control treatments, and the relative reductions of insulin Cmax were calculated to be 24% after a 225 g serving [18] and 31% after a 297 g serving of lentils [19]. The reduction of insulin Cmax appears to be independent of lentil serving, as reductions of similar magnitude were seen with both low and high lentil serving sizes.

**Table 1:** Summary of data obtained from studies involving healthy subjects.

			Lentil Treatment					Control Treatment					Outcomes						
Study	Participants		Lentil Serving Size (g)	Protein (g)	DF (g)	CHOav (g)	TC (g) ^†^	Control Serving (g)	Protein (g)	DF (g)	AC (g)	TC (g) ^†^	BG Cmax Lentil Group	BG Cmax Control Group	Relative Difference in BG Cmax (%)	BG AUC Lentil Group (mmol × min/L)	BG AUC Control Group (mmol × min/L)	Relative Difference in BG AUC (%)	BG AUC Time Period
Akhtar, Asim, and Wolever 1987 [16]	14 males aged 35.2 ± 1.54 years, BMI 23.6 ± 0.55 kg/m^2^		89.3	24.6	4.5	-	25	110	15.1	1.5	-	50	4.88 @ 60 min	5.22 @ 120 min	6.51	35.5	79.4	55.3	3 h
Anderson, Liu, Smith, Liu, Nunez, Mollard and Luhovyy, 2014 [20]	males aged 18–30 years, normal BMI		405.5	16.5	11.3	38.7	50	405.5	10.5	7.9	38.7	46.6	7.1 @ 30 min	7.7 @ 30 min	7.79	97.1	125.5	22.6	2 h
Bennett, Chilibeck, Barss, Vatanparast, Vandenberg and Zello 2012 [21]	14 recreational soccer players, aged 22–27 years, BMI 22 kg/m^2^		444	36	24	105	129	436	16	7	92	99	7.0 @ 15 min	9.0 @ 15 min	22.22	-	-	-	-
Jenkins, Ghafari, Wolever, Taylor, Jenkins, Barker, Fielden and Bowling, 1982 [22]	17 adults aged 28 ± 2 years		297.5 *	22	11	-	50	120	10.5	10.2	-	50	-	-	-	60	173	65.3	2 h
Jenkins, Wolever, Taylor, Griffiths, Krzeminska, Lawrie, Bennett, Goff, Sarson and Bloom, 1982 [10]	7 adults aged 26 ± 3 years		715 *	57	29	-	127	280	57	26	-	128	5.1 @ 30 min	7.3 @ 30 min	30.14	49	178	72.5	2 h
MacPherson 2018 [11]	adults aged 18–40 years, BMI 20–30 kg/m^2^	male	100	9.88	5.55	13.8	26.3	54.6	2.23	1.07	15.5	13.6	5.0 @ 30 min	5.7 @ 30 min	12.28	30	65	53.9	2 h
		female	50	4.94	2.78	6.91	13.2	27.3	1.16	0.56	8.08	7.1	4.7 @ 15 min	5.1 @ 15 min	7.84	20	30	33.3	2 h
			**Lentil Treatment Comparison**					**Control Treatment Comparison**					**Outcomes**						
**Study**	**Participants**		**Lentil Serving Size (g)**	**Protein (g)**	**DF (g)**	**CHOav (g)**	**TC (g) ^†^**	**Control Serving (g)**	**Protein (g)**	**DF (g)**	**AC (g)**	**TC (g) ^†^**	**BG Cmax Lentil Group**	**BG Cmax Control Group**	**Relative Difference in BG Cmax (%)**	**BG AUC Lentil Group (mmol × min/L)**	**BG AUC Control Group (mmol × min/L)**	**Relative Difference in BG AUC (%)**	**BG AUC Time Period**
Mollard, Wong, Luhovyy and Anderson, 2011 [23]	25 males aged 20–30 years, BMI 20.0–24.9 kg/m^2^		332.9	29.1	18.3	98.7	117	446.5	22.8	2.8	100.4	103.2	8.0 @ 40 min	8.2 @ 40 min	2.44	392.9	431.3	8.9	260 min
Mollard, Wong, Luhovyy, Cho and Anderson, 2014 [24]	15 males aged 18–35 years		332.9	18.3	13.5	52.2	65.7	116.6	9.9	2.8	64	66.8	7.5 @ 30 min	9.0 @ 30 min	16.67	133.8	239.7	44.2	135 min
Ramdath, Wolever, Siow, Ryland, Hawke, Taylor, Zahradka and Aliani, 2018 [15]	20 males and females, 18–75 years, BMI < 30 kg/m^2^		321.8	25.3	22	50.1	72.1	68.1	5	4.6	50	54.6	4.8 @ 30 min	8.2 @ 30 min	41.46	65	144	54.9	2 h
Tovar, Granfeldt and Bjorck, 1992 [25]	10 adults aged 36 ± 2.5 years, BMI 22.4 ± 0.9 kg/m^2^		221.52 *	17.6	11.1	30	41.1	70	19	1.4	30	31.4	6.0 @ 30 min	6.7 @ 30 min	10.45	-	-	-	2 h
Wolever, Jenkins, Ocana, Rao and Collier, 1988 [17]	5 adults aged 24 ± 0.3 years		3.2 g/kg, raw weight	45.7	22.5	102.1	124.6	1.7 g/kg	45.6	0	102	102	5.0 @ 45 min	7.0 @ 60 min	28.57	16	88	81.8	2 h
Wong, Mollard, Zafar, Luhovyy and Anderson, 2009 [26]	14 men aged 18–35 years, BMI 20–25 kg/m^2^		451	18.3	13.5	52.2	65.7	235	9.9	2.8	64	66.8	7.3 @ 15 min	8.5 @ 15 min	14.14	96.3	175.4	45.1	2 h

DF, Dietary Fibre; CHOav, Available Carbohydrates; TC, Total Carbohydrates; BG, blood glucose; AUC, area under the curve. * Raw weights provided by these studies were converted to cooked weights using the average moisture content of green lentils (68.4%), as gathered from previous research (unpublished data). The cooked weight was calculated as follows: (dry weight/% solids) × 100, where % solid = 100–68.4. ^†^ Study did not include TC, AC and DF were summed to provide total carbohydrates value.

**Table 2:** Summary of data obtained from studies of subjects with T2D.

		Lentil Treatment					Control Treatment					Outcomes					
Study	Participants	Lentil Serving Size (g)	Protein (g)	DF (g)	CHOav (g)	TC (g) ^†^	Control Serving (g)	Protein (g)	DF (g)	AC (g)	TC (g) ^†^	BG Cmax Lentil Group	BG Cmax Control Group	BG AUC Lentil Group (mmol× min/L)	BG AUC Control group (mmol× min/L)	Relative Difference in BG AUC (%)	BG AUC Time Period
Akhtar 1987 [16]	14 adults aged 52.5 ± 2.46 years, BMI 24.3 ± 1.13 kg/m^2^	89.3	24.6	4.5	-	25	110	15.1	1.5	-	50	11.2 @ 30 min	14.0 @ 30 min	95.5	215.5	55.7	3 h
Bornet, Costagliola, Rizkalla, Blayo, Fontvieille, Haardt, Letanoux, Tchobroutsky and Slama, 1987 [19]	18 adults aged 57 ± 2 years, BMI 27.9 ± 1.1 kg/m^2^	225	20.5	3.25	50	53.25	-	-	-	50	50	2.1 @ 120 min ^†^	6.1 @ 60 min ^†^	-	-	-	-
Coulston, Hollenbeck, Liu, Williams, Starich, Mazzaferri and Reaven, 1984 [27]	8 adults aged 59 ± 2 years, BMI 27.3 ± 0.6 kg/m^2^	49.9 **	12.3	5.8	30	35.8	-	3.7	4.1	38.2	34.1	13.7 @ 120 min ^†^	17.5 @ 120 min ^†^	2065 ^‡^	2731 ^‡^	24.4	3 h
Jenkins, Wolever, Jenkins, Thorne, Lee, Kalmusky, Reichert and Wong, 1983 [28]	12 adults aged 67 ± 2 years, 77.1 ± 4.4 kg	297.5 *	22.4	11	-	50	-	22.1	7.3	-	51.2	3.0 @ 120 min	6.2 @ 90 min	359	806	55.5	3 h
Jenkins, Wolever, Taylor, Ghafari, Jenkins, Barker and Jenkins, 1980 [29]	6 adults aged 43 ± 5 years	129.8 *	38.8	10	-	42.5	-	35.5	9.3	-	43.9	1.1 @ 30 min	4.7 @ 60 min	-	-	-	-
Krezowski, Nuttall, Gannon, Billington and Parker, 1987 [18]	8 male untreated diabetics aged 65 ± 2 years	297.5 *	22.4	11	-	50	-	-	-	-	50	1.9 @ 120 min ^†^	7.4 @ 60 min ^†^	380 ^‡^	1176 ^‡^	67.7	5 h

DF, Dietary Fibre; CHOav, Available Carbohydrates; TC, Total Carbohydrates; BG, blood glucose; AUC, area under the curve. * Raw weights provided by these studies were converted to cooked weights using the average moisture content of green lentils (68.4%), as gathered from previous research (unpublished data). The cooked weight was calculated as follows: (dry weight/% solids) × 100, where % solid = 100–68.4. ** Unspecified if wet or dry weight. ^†^ TC was calculated by adding AC and DF if the study did not state its own TC value. AC only included if value given in study. ^‡^ Values were converted from mg × h/dL to mmol × min/L using the conversion factors, 1 mg/dL glucose = 0.05551 mmol/L glucose and 1 h = 60 min. ^†^ Values were converted from mg/dL to mmol/L using the conversion factor, 1 mg/dL glucose = 0.05551 mmol/L glucose.

**Table 3:** Summary of studies that assessed blood insulin after lentil consumption in either healthy or diabetic subjects.

		Lentil Treatment				Control Treatment				Outcomes						
Study	Participants	Lentil Serving Size (g)	Protein (g)	DF (g)	TC (g)	Control Serving (g)	Protein (g)	DF (g)	TC (g)	Insulin Cmax Lentil Group	Insulin Cmax Control Group	Relative Difference Insulin Cmax (%)	Insulin AUC Lentil Group	Insulin AUC Control Group	Relative Reduction Insulin AUC (%)	Insulin AUC Time Period
Bennett, Chilibeck, Barss, Vatanparast, Vandenberg and Zello 2012 [21]	14 recreational soccer players aged 22–27 years, BMI 22 kg/m^2^	444	36	24	129	436	16	7	99	135 pmol/L	125 pmol/L	8.0	-	-	-	-
Bornet, Costagliola, Rizkalla, Blayo, Fontvieille, Haardt, Letanoux, Tchobroutsky and Slama, 1987 [19]	18 type 2 diabetics	225	20.5	3.25	50		-	-	50	216 pmol/L ^†^	283 pmol/L ^†^	23.7	-	-	-	-
Jenkins, Wolever, Taylor, Griffiths, Krzeminska, Lawrie, Bennett, Goff, Sarson and Bloom, 1982 [10]	Healthy adults (5 men, 2 women)	715 *	57	29	156	280	57	26	154	100 pmol/L	230 pmol/L	56.5	-	-	-	-
Krezowski, Nuttall, Gannon, Billington and Parker, 1987 [18]	8 male untreated diabetics	297 *	22.4	11	50	120	10.6	10.2	50	174 pmol/L ^†^	251 pmol/L ^†^	29.7	44 µU×h/mL	91 µU × h/mL	51.7	5 h
MacPherson 2018 [11]	Healthy adult males and females aged 18–40 years, BMI 20–30 kg/m^2^	100	9.88	5.55	26.3	54.6	2.23	1.07	13.6	50 pmol/L	80 pmol/L	37.5	1100 nmol × min/L	2000 nmol × min/L	45	2 h
Tovar, Granfeldt and Bjorck, 1992 [25]	10 healthy adults aged 36 ± 2.5 years, BMI 22.4 ± 0.9 kg/m^2^	70	17.6	11.1	41.1	70	19	1.4	31.4	240 pmol/L	270 pmol/L	11.11	-	-	-	-

DF, Dietary Fibre; TC, Total Carbohydrates; AUC, area under the curve. * Raw weights provided by these studies were converted to cooked weights using the average moisture content of green lentils (68.4%), as gathered from previous research (unpublished data). The cooked weight was calculated as follows: (dry weight/% solids) × 100, where % solid = 100–68.4. ^†^ Insulin Cmax values were converted from µU/mL to pmol/L using the conversion factor 1 µU/mL = 6 pmol/L (Diabetes Care 1998). mU/mL was converted to pmol/L using the conversion factor of 1mU/L = 6 pmol/L (Vølund 1993). (Krezowski et al. provided an original value of 10.5 µU/mL and 23 µU/mL. for lentil and control treatments, respectively. Bornet et al. gave an original value of 25 mU/L and 42 mU/L for lentil and control treatments, respectively.

## 4. Discussion

This review provided several insights into the hypoglycemic effects of lentil consumption using a limited number of available studies involving both healthy and diabetic participants. Pooled data from these studies suggest that lentil serving sizes higher than 100 g cooked weight do not lead to further reductions in BG AUC in healthy participants. Relative reductions in BG AUC and BG Cmax were greater with increased levels of protein and dietary fibre content, as noted in cooked lentil servings of 715 g (57 g protein, 29 g dietary fibre) [10] and 3.2 g lentils/kg of participant body weight (average 45.7 g protein, 22.5 g dietary fibre/serving) [17]. These larger serving sizes had weaker correlations between CHOav and reductions in BG AUC or BG Cmax. The amount of CHOav proved to be a highly variable factor in relative reduction in BG AUC, as lentil servings with high CHOav (~100 g) displayed both high and low reductions. A lentil treatment with 102.1 g of CHOav that was associated with a greater relative reduction in BG AUC (82%) had higher protein content. Additionally, relative reductions in BG Cmax followed a similar pattern: increased protein (25.3–57 g) and dietary fibre (22–29 g) content led to greater reductions. Again, the impact of CHOav was difficult to interpret, as the relative reduction in BG Cmax associated with the amount of CHOav content was not consistent across the studies examined. Larger servings of lentils, leading to higher levels of protein and dietary fibre, may be optimal for achieving a maximum reduction in BG AUC and Cmax in healthy participants. 

The hypoglycemic effect of lentils has been documented frequently in the literature, yet the effect size and lentil dosage vary greatly between studies and serving–response studies are scarce. Cumulative evidence from several studies has shown that BG AUC was lower in lentil groups when equivalent amounts of protein [10,17], dietary fibre [22], CHOav [15,17,20,25] and serving sizes [20] of control treatments were matched. This trend is in alignment with previous research in which lentils were found to have a low glycemic index compared to high-starch containing foods, thereby attenuating large fluctuations in BG [30,31]. The lack of a clear dose–response observed in this review indicates that both low and high serving sizes of lentils have similar effects on BG. At a relatively low serving size of 100 g of cooked green lentils, there was a 54% relative reduction in BG AUC compared to control [11]. Health Canada guidelines state that a minimum of 20% postprandial glucose reduction is significant [32]. Our analysis shows that consuming even a relatively small serving size of lentils (50–100 g) seems to confer this benefit. Consumers are more likely to eat smaller quantities of lentils [33], and the current trend suggests that they would continue to experience the BG attenuation effects of lentils at these lower serving sizes. In contrast to glucose, insulin Cmax differed between low and high lentil servings. We found that both healthy and diabetic participants had lower insulin Cmax with lentil treatment compared to control, as outlined in Table 3. As an example, healthy participants who consumed 715 g of cooked green lentils had an insulin Cmax of 100 pmol/L [10], compared to an insulin Cmax of 50 pmol/L with 100 g of cooked green lentils [11]. The higher serving size was associated with a 72% relative reduction in BG AUC, whereas the 100 g serving led to a 54% relative reduction compared to control. This data suggests that the hypoglycemic effect of lentils is not linear, and is not due to an increase in blood insulin levels [11,33]; this is an important finding since hyperinsulinemia is a hallmark of T2D [34].

Studies have used lentils in different forms and found that while processing can have an effect on the degree to which lentils lower BG, results from our analysis indicate that processed lentils also lowers BG AUC, compared to control treatments [15,20]. These studies suggest that consuming lentils in different forms (whole, powder, flour) maintains the BG AUC lowering property when compared to starchy controls. A study that examined the glycemic response of whole, puréed, and powdered green lentils, found that the pre and postprandial absolute glucose concentration in participants consuming these lentil preparations were lower than the control (whole wheat flour) overall [20]. In addition, all treatments in the Anderson et al. (2014) study had lower preprandial BG AUC compared to the control diet; whole and powdered lentils had a statistically lower preprandial BG AUC (*p* < 0.05) compared to the control. Puréed lentils also had the lowest postprandial BG AUC compared to the control with a difference of 31.3 mmol × min/L, although none of the postprandial differences were reported to be significant [20]. Similar to lentil powder, another study that incorporated 20% lentil flour into baked muffins observed a relative reduction in BG AUC of 25% after 2 h [35]. This result falls within the range of relative reductions in BG AUC for the whole lentil studies (22.81% to 81.82%; Table 1), and despite the change in preparation, the reduction of BG AUC is still above the important threshold of 20% [32]. This finding suggests that the components responsible for the glucose-lowering action of lentils remain biologically active through both processing and baking. Unfortunately, a single lentil flour study does not provide enough evidence to conclude that lentils have the same effect regardless of how they are prepared and consumed. More human studies need to be carried out to determine if and how the form in which lentils are consumed changes the glycemic response in healthy and diabetic participants.

While lentils are known to reduce BG, the specific component responsible for this effect continues to be debated. Some studies have suggested that it is the protein content of a meal that lowers BG by increasing insulin secretion [36], referring to dietary protein as a “potent insulin secretagogue” [37]. However, the available studies show that those consuming the highest lentil serving sizes had the lowest insulin levels, therefore an increase in insulin is unlikely to be the primary factor in the glycemic effect of lentils. There is another potential mechanism by which proteins may reduce BG. Protein in lentils could lead to protein–starch interactions that block digestive enzymes from accessing starch, thereby reducing the amount of glucose available for absorption through the intestines [38]. Although unlikely, it was suggested that products of protein breakdown, such as small peptide chains, could lower BG through competitive inhibition of intestinal enzymes and prevent the breakdown of starch to glucose. These smaller peptide chains were shown to competitively inhibit enzymes responsible for breaking down insulin, thereby permitting insulin to persist longer and continue to lower the BG level while remaining at a lower concentration itself [39]. It has also been shown that lentil polyphenols are able to significantly inhibit α-glucosidase, a key enzyme in the digestion of dietary carbohydrates; this could partially account for the BG lowering effect of lentils [5]. Wolever et al. (1988) created a red lentil treatment that contained the same amount of protein content as the control diet (45 g), which resulted in significant BG responses, including reduced peak rise, BG AUC, and mean postprandial BG concentrations. The implications of the matched protein content study suggest that the BG lowering responses may be due to other differences in digestion and absorption rates, and are not contingent on protein content alone [17]. Based on the data analysed in this review, we determined that maximum relative reductions in BG AUC are observed at 45–57 g of protein content in the lentil treatments. Proteins certainly appear to have a role in the health benefits associated with consuming lentils; however, the mechanisms or combination of mechanisms that allow proteins to reduce BG is still unknown.

Studies analysing the impacts of dietary fibre on BG have produced varying results. A 2012 meta-analysis found that fibre supplementation of 4–42 g/d reduced fasting BG by 0.85 mmol/L (95% CI, 0.46–1.25) in participants with T2D when compared to placebo treatment [40]. A wide range of fibre servings was provided to participants during acute feeding trials, with 15 g/d being the most common serving [40]. Through our analyses, we determined that maximum relative reductions in BG AUC are observed at 22–30 g of dietary fibre content in the lentil treatments. Foods with high amounts of soluble viscous fibre, such as oat gum, significantly reduce BG and insulin relative to its viscosity [41]; however, less viscous soluble fibres such as pectin and psyllium were ineffective [42]. The largest relative reduction in BG AUC (81.8%) observed during our analysis was from a study in which only the amount of dietary fibre differed between the control (0 g) and lentil treatments (22.5 g), while protein, CHOav and energy content were matched [17]. However, the second-largest reduction in BG AUC (72.5%) had a control and lentil treatment that differed by only 3 g in dietary fibre content [10]. Taken together, the dietary fibre content in a meal likely has a role in reducing BG AUC, but the amount of dietary fibre needed for this effect and the associated mechanism(s) of action is yet to be determined.

Of the two macronutrients and dietary fibre examined, CHOav was expected to elicit a greater BG AUC as CHOav content increased, and this was observed from the available data. Like many studies, protein and dietary fibre showed U-shaped quadratic relationships with BG AUC, whereas CHOav displayed a linear relationship in our analyses. Similarly, a study involving untreated T2D participants reported that BG response increased linearly as the amount of ingested carbohydrates increased [43]. Results from the studies reviewed here show that the absolute BG AUC, BG Cmax, and the relative reductions of BG AUC and BG Cmax of healthy participants receiving lentil treatments are only weakly correlated with the amount of CHOav, and more moderate correlations in these measures were observed with dietary fibre. There were inconsistencies between how CHOav was reported in the collected literature, as some studies only mentioned total carbohydrates, while others were unclear as to whether the measurement was total carbohydrates or CHOav. 

It was observed that dietary fibre content follows similar trends to protein in reducing BG AUC, and likely has an important role in its reduction. Evidence gathered in this review suggests that the amount of protein in a lentil treatment has a stronger influence on reducing BG AUC relative to control when compared to the amount of dietary fibre or CHOav; this relationship was previously examined through an assessment of glycemic index in combination with protein and total dietary fibre [44]. The GI of different foods was found to be associated with its protein and dietary fibre content [44,45], and our analysis indicates a moderate correlation with protein and relative reductions in BG AUC, as well as a strong correlation with dietary fibre with relative reductions in BG Cmax compared to controls. However, it was also suggested that the correlation between GI, protein, fibre, and fat is due to the fact that a large number of studies utilise legumes, which contain more of these macronutrients and dietary fibre when compared to other foods [45]. Furthermore, it was previously argued that while correlated, protein does not cause lower glycemic indices, and glycemic responses to foods cannot be predicted from their macronutrient composition since GI is a feature of the carbohydrates found in food [44].

## 5. Summary

The studies analysed in this review provide support for the ability of lentil consumption to lower BG in participants with or without T2D. This beneficial effect of consuming lentils is likely due to their complex macronutrient content [9,44]. Both protein and dietary fibre content were identified as potential factors in the glycemic response of lentils, however, the direct evidence for either remains inconclusive. Our analysis suggests a range of 45–57 g of protein and 22–30 g of dietary fibre content in lentil treatments are needed to obtain a maximum relative reduction in BG AUC, while the CHOav content was found to have only a weak relationship with relative reductions of BG AUC. Both high (>100 g) and low (<100 g) lentil serving sizes were assessed for BG AUC lowering; there were moderate correlations between serving and BG AUC and between serving and relative reductions in BG Cmax. Although increased lentil serving sizes moderately influenced these parameters, no clear relationship was found between serving and relative reductions in BG AUC, making it difficult to predict a minimal serving size for an optimal and consistent glycemic benefit. Another important aspect of this research is the potential that the beneficial effects of lentils may be due to an interaction with insulin. In studies that reported insulin levels, the insulin area under the curve was lower for lentil meals compared to controls, and this insinuates that lentil consumption has a role in mediating insulin levels. The relationship between lentil consumption and insulin response is a topic that requires more research in order to draw firm conclusions. Persons with T2D have the greatest potential to benefit from this research as BG and insulin control are central to the management of this condition. Overall, the studies examined for this review corroborate findings that lentil consumption provides beneficial effects on both BG and insulin levels, although further investigation is required to fully understand how lentils and other pulses are contributing to this lowering effect.
